# Nicotinamide Promotes Adipogenesis in Umbilical Cord-Derived Mesenchymal Stem Cells and Is Associated with Neonatal Adiposity: The Healthy Start BabyBUMP Project

**DOI:** 10.1371/journal.pone.0159575

**Published:** 2016-07-14

**Authors:** Allison L. B. Shapiro, Kristen E. Boyle, Dana Dabelea, Zachary W. Patinkin, Becky De la Houssaye, Brandy M. Ringham, Deborah H. Glueck, Linda A. Barbour, Jill M. Norris, Jacob E. Friedman

**Affiliations:** 1 Department of Epidemiology, Colorado School of Public Health (CSPH), Aurora, Colorado, United States of America; 2 Department of Pediatrics, University of Colorado School of Medicine (UC-SOM), Aurora, Colorado, United States of America; 3 Department of Epidemiology, CSPH, Aurora, Colorado, United States of America; 4 Department of Biostatistics & Informatics, CSPH, Aurora, Colorado, United States of America; 5 Department of Biochemistry & Molecular Genetics, UC-SOM, Aurora, Colorado, United States of America; 6 Department of Medicine, Division of Endocrinology, Metabolism, and Diabetes, UC-SOM, Aurora, Colorado, United States of America; 7 Department of Obstetrics and Gynecology, Division of Maternal-Fetal Medicine UC-SOM, Aurora, Colorado, United States of America; University of Catania, ITALY

## Abstract

The cellular mechanisms whereby excess maternal nutrition during pregnancy increases adiposity of the offspring are not well understood. However, nicotinamide (NAM), a fundamental micronutrient that is important in energy metabolism, has been shown to regulate adipogenesis through inhibition of SIRT1. Here we tested three novel hypotheses: 1) NAM increases the adipogenic response of human umbilical cord tissue-derived mesenchymal stem cells (MSCs) through a SIRT1 and PPARγ pathway; 2) lipid potentiates the NAM-enhanced adipogenic response; and 3) the adipogenic response to NAM is associated with increased percent fat mass (%FM) among neonates. MSCs were derived from the umbilical cord of 46 neonates born to non-obese mothers enrolled in the Healthy Start study. Neonatal %FM was measured using air displacement plethysmography (Pea Pod) shortly after birth. Adipogenic differentiation was induced for 21 days in the 46 MSC sets under four conditions, +NAM (3mM)/–lipid (200 μM oleate/palmitate mix), +NAM/+lipid, –NAM/+lipid, and vehicle-control (–NAM/–lipid). Cells incubated in the presence of NAM had significantly higher PPARγ protein (+24%, p <0.01), FABP4 protein (+57%, p <0.01), and intracellular lipid content (+51%, p <0.01). Lipid did not significantly increase either PPARγ protein (p = 0.98) or FABP4 protein content (p = 0.82). There was no evidence of an interaction between NAM and lipid on adipogenic response of PPARγ or FABP4 protein (p = 0.99 and p = 0.09). In a subset of 9 MSC, SIRT1 activity was measured in the +NAM/-lipid and vehicle control conditions. SIRT1 enzymatic activity was significantly lower (-70%, p <0.05) in the +NAM/-lipid condition than in vehicle-control. In a linear model with neonatal %FM as the outcome, the percent increase in PPARγ protein in the +NAM/-lipid condition compared to vehicle-control was a significant predictor (β = 0.04, 95% CI 0.01–0.06, p <0.001). These are the first data to support that chronic NAM exposure potentiates adipogenesis in human MSCs *in-vitro*, and that this process involves PPARγ and SIRT1.

## Introduction

Nutrition in pregnancy has been shown to affect various aspects of fetal growth and development [1]. A maternal high-fat diet (HFD) during pregnancy increases newborn adiposity in both rodents and in non-human primates [2–4]. In addition to increased macronutrient content, modulation of adipogenesis may also occur through pathways involving micronutrients that can interact directly with proteins involved in promoting adipose tissue accretion. Evidence from cell culture models suggests that niacin may be one such “obesogenic” micronutrient, where bone marrow-derived mesenchymal stem cells (MSCs) treated with nicotinamide (NAM), the amide form of niacin, demonstrated increased adipogenesis in vitro [5]. Further, niacin treatment has recently been linked to increased risk of new onset diabetes in a large meta-analysis of clinical trials [6]. However, the mechanism by which niacin induces a greater adipogenic response and metabolic dysfunction is unclear.

One prominent nutrient sensor that governs adipogenesis is the silent mating type information regulation 2 homolog 1 (SIRT1), a NAD(+)-dependent deacetylase. SIRT1 has been shown in several *in-vitro* studies to govern adipogenesis in a 3T3L pre-adipocyte cell model [7]. In addition, SIRT1 governs adipogenesis *in-vivo*. Adipose-specific SIRT1 knockout mice have increased PPARγ protein activity, increased adiposity, and metabolic dysfunction [8]. SIRT1 has also been shown to inhibit adipogenic differentiation when stimulated by a pharmacologic activator, resveratrol [9]. Importantly, SIRT1 is potently inhibited by NAM suggesting that nutritional micronutrient levels govern its activity. Likewise, HFD inhibits adipose tissue SIRT1 activity through proteolysis [10], and its activity is suppressed in the fetal livers of non-human primate offspring whose mothers consumed a HFD during gestation [11]. Taken together, these data suggest a pathway whereby dietary fat and NAM promote adipogenesis, possibly through modulation of a SIRT1 and adipogenic proteins (e.g. PPARγ and FABP4). Moreover, NAM-mediated inhibition of SIRT1 activity may be one mechanism by which maternal diet in pregnancy could increase adipogenesis in-utero and fetal adiposity.

Micronutrients have received very little attention as modulators of fetal developmental programming. In this study we investigate the effect of nicotinamide, in combination with excess fatty acids, on SIRT1 activity and the adipogenic response potential of human umbilical cord-derived mesenchymal stem cells, which represent a fetal stem cell population. We hypothesized that *in-vitro* NAM exposure would decrease SIRT1 activity and induce greater adipogenic response and that co-incubation with lipids would amplify these effects. We also tested the hypothesis that NAM-induced increases in adipogenic response (e.g. increased protein levels of PPARγ and FABP4) would be associated with infant adiposity at birth, possibly reflecting the potential for NAM exposure in-utero to increase adipogenic response in fetuses across a range of fat mass.

## Materials and Methods

We cultured human umbilical cord-derived mesenchymal stem cells (MSCs) from umbilical cord tissue collected from neonates whose mothers were enrolled in the Healthy Start study, a longitudinal pre-birth cohort study of ethnically diverse women in Colorado. The Healthy Start study recruited pregnant women ages 16 and older with a gestational age less than 24 weeks from the obstetrics clinics at the University of Colorado Hospital during 2010–2014. Women were excluded if they had prior diabetes, a prior premature birth or fetal death, asthma with active steroid management, serious psychiatric illness, or a current multiple pregnancy. The Healthy Start study was approved by the Colorado Multiple Institutional Review Board and all participants provided written informed consent prior to delivery for collection of the umbilical cord tissue for cell culture purposes (COMIRB #09–0563).

Healthy Start data collection methods have been described in detail elsewhere [12]. Briefly, with regard to measures used in this study, infant body composition was measured within 72 hours after birth using air displacement plethysmography (Pea Pod). Fat mass and fat-free mass were estimated from total body mass and percent fat mass (%FM) was generated by calculating the proportion of fat mass over total body mass. Body composition was measured twice for each infant with a third measurement taken if the two preceding %FM measures differed by more than two percentage points. Values used in this study are the average of the two closest measures of body composition.

### Sample Collection, MSC Isolation and Culture

We collected, cultured, and cryogenically stored 165 samples of unique MSC sets from infants that make-up the Healthy Start Baby Biology of intra-Uterine Metabolic Programming (BabyBUMP) Project, which is the mechanistic arm of the parent Healthy Start study. For the current study, MSCs from up to 46 individual infants born to non-obese, healthy mothers were randomly selected for experimentation (28 with BMI < 25 kg/m^2^, 18 with BMI 25–25.9 kg/m^2^). These 46 MSC sets (cell passage 3) were used to measure cellular protein content of adipogenic response markers, PPARγ and FABP4 (detailed in the section entitled Adipogenic Induction of MSCs). A subset of 9 MSC sets (cell passage 4) was selected for analysis of SIRT1 enzymatic activity and additional measures (detailed in the sections entitled SIRT1 Activity Assay, Acetylation of SIRT1 Target Proteins, and Quantitative PCR Analysis). This subset of 9 was selected from infants born to normal weight (BMI < 25 kg/m2) mothers and had protein profiles representative of the adipogenic response.

At delivery, trained research personnel cut a 4-inch section of the umbilical cord below the placement of the clamp and rinsed the segment thoroughly with deionized water. The tissue section was then immersed in PBS with 0.01% penicillin and streptomycin (PEN-STREP) and stored at 4°C until processing. All tissue samples were processed within 24 hours of delivery.

Umbilical cord tissue was cut into 12, 50–100 mg pieces with 6 pieces arranged equidistant, Wharton’s Jelly side down, on each of two 10 cm dishes with bovine serum albumin (BSA) (Sigma-Aldrich, St. Louis, MO, USA) to assist with adhesion of the tissue to the plate. Tissue was immersed in low-glucose (1.0 g/L) Dubelcco’s Modified Eagle’s Serum (DMEM) supplemented with MSC growth factors (MSCGM;Lonza, Walkersville, MD, USA). Cells were then cultured to 80–100% confluence in MSCGM at which point they were stored in liquid nitrogen with 0.05% DMSO for cryogenic preservation. We have shown this method to yield cells >98% positive for MSC markers [13].

### Adipogenic Induction of MSCs

For adipogenesis experiments, cells were thawed and sub-cultured to confluence into 10 cm dishes. At 100% confluence, cells were plated at an approximate cell density of 1x10^4^/cm^2^ onto standard 96-well culture plates. Day 0 of experimentation occurred when experimental plates were 90–100% confluent. Differentiation was induced as described [12]. Briefly, on Day 0 growth media was replaced with adipogenic induction media (AIM) consisting of low-glucose DMEM supplemented with 5% fetal bovine serum (FBS), 0.01%-STREP, 1.0 uM dexamethasone (DEX), 0.2 mM indomethasone (INDO), and 170 pM insulin [14]. After 3 days of AIM, media was changed to adipogenic maintenance media (AMM) consisting of low-glucose DMEM, 5% FBS, 10 units/mL:10 ug/mL PEN:STREP, and 170 nM insulin and incubated for 3 days. Cells were exposed to AIM twice more with a rest period of AMM in between (S1 Table).

For adipogenesis in the presence of NAM and/or lipid, adipogenic induction was performed as described above, with or without 3 mM NAM and/or lipid (1 mM carnitine + 200 μM oleate and palmitate in a 2:1, oleate:palmitate ratio, bound to BSA at a molar concentration of 2.5:1). The four treatment conditions are: -NAM/-lipid (vehicle-control), +NAM/-lipid, -NAM/+lipid, and +NAM/+lipid. The oleate-to-palmitate ratio that we chose for our lipid exposure condition is most representative of the physiological lipid composition obtained from the diet [15]. Further, the concentration of the lipid is relatively low to limit toxicity to the cells [15]. Prior to experimentation, a dose-response curve of NAM (2.5, 3.0, 4.5 mM) was performed to determine the optimal concentration for PPARγ induction (S1 Fig). Ethanol was used as vehicle-control. On day 21 plates were rinsed twice with PBS and then fixed to culture plates with 4% formalin diluted in PBS for 5 min at room temperature. Plates were stored in PBS at 4°C until assays were performed.

### In-Cell ELISA (ICE)

#### Antibody Selection and Optimization

We quantified adipogenic differentiation response by measuring protein content of PPARγ (Cell Signaling Technology, Inc., Danvers, MA, USA) and FABP4 (Cell Signaling). We also measured intracellular SIRT1 protein content (Santa Cruz Biotechnology, Inc., Dallas, TX, USA). Beta(β)-actin was measured for total protein normalization (Cell Signaling). Primary antibodies for these proteins were selected based on their successful application by the manufacturer to either immunohistochemistry or in-cell ELISA platforms. Goat anti-rabbit horseradish peroxidase (HRP)-conjugated secondary antibody (Goat≠Rabbit; Abcam, Cambridge, MA, USA) was also selected based on this criterion.

Antibody optimization for the ICE was performed on purchased human bone marrow-derived mesenchymal stem cells (hBM-MSC; Lonza). hBM-MSCs were grown in 96-well plates for 21 days according to the adipogenic differentiation protocol above (without treatment conditions). Antibody dilutions were optimized in-house, listed in S2 Table. The slope profiles for each antibody dilution were analyzed. Dilutions presenting with multiple stable slope values over 5 minute intervals within the 30-minute read time were chosen as optimal dilution factors for the respective antibody.

#### ICE Assay Procedure

Cells were permeabilized at room temperature rotating for 1 hour using permeabilizaton/blocking buffer (0.1% Fraction V BSA, 5% goat serum, 0.3% Triton X-100, and 0.2% sodium azide in PBS). Wells were rinsed at room temperature for 2x5 minutes with rotation with rinse buffer (0.01% Fraction V BSA and 1% goat serum in PBS). Primary antibodies were diluted in antibody buffer (0.1% Fraction V BSA and 5% goat serum in PBS) and added to all wells, except the no primary control (NPC) wells. Plates were incubated overnight at 4°C with rotation.

After primary antibodies were removed, wells were rinsed at room temperature for 7x5 minutes with rotation using rinse buffer. Goat≠Rabbit secondary antibody (S2 Table) was added to all wells and plates were incubated at room temperature for1 hour with rotation. Wells were then rinsed at room temperature for 3x5 minutes with rotation using rinse buffer, then for 2x5 minutes with rotation using PBS. Wells were emptied and blotted dry on absorbent paper, and HRP developing solution (Thermo Fisher Scientific Inc., Waltham, MA, USA) was added to all wells. Color change was measured at 1-minute intervals for 10 min at 650 nm. Stable slopes for 5 consecutive minutes were used to quantify the content of proteins of interest. All data were normalized to β-actin protein content.

### Lipid Accumulation and Oil-Red-O Staining

Intracellular lipid accumulation in differentiated cells on d21 was measured on cells fixed in 4% formalin and stained with 0.2% Oil Red-O dissolved in 85% propylene glycol for one hour. Cells were rinsed with 85% propylene glycol (2x) followed by two rinses with deionized water. Pictures were then taken of representative plate wells using a phase contrast microscope with a10x objective lens. The ORO stain was then solubilized from the cell by submergence in isopropanol for 5 minutes. Lipids were measured on a spectrophotometer (520 nm) with endpoint analysis and normalized to total protein.

### SIRT1 Activity Assay

Cells were grown to confluence in 6 cm petri dishes, differentiated for 9 days (peak adipogenesis in the second AIM induction) and 21 days (endpoint of adipogenic induction) using the adipogenic protocol with and without NAM treatment and harvested in CellLytic lysis buffer (Sigma-Aldrich) with 0.01% protease inhibitor (Sigma-Aldrich) added. Cell lysate was sonicated and centrifuged; the supernatant was flash frozen and stored at -80°C until assay.

SIRT1 protein was extracted from cell supernatant using immunoprecipitation. Briefly, a 1:50 dilution of SIRT1 antibody (Abcam) was added to each sample of cell supernatant and incubated overnight at 4°C with agitation. The antibody-supernatant mixture was added to a 50% slurry of protein A agrose bead (Cell Signaling) and incubated for 3 hours at 4°C with agitation. SIRT1 protein activity was then measured by fluorometric assay at 350 nm (Abcam) according to the manufacturer’s protocol for quantification of SIRT1 activity.

Total SIRT1 protein was measured simultaneously by Simple Western size-based protein assay (WES, ProteinSimple, Santa Clara, CA) following manufacturer’s protocol. Results from WES were analyzed using ProteinSimple Compass software. SIRT1 activity in each cell set was normalized to the cell set’s total SIRT1 protein as measured by WES.

### Acetylation of SIRT1 Target Proteins

PPARγ and β-catenin are directly de-acetylated by SIRT1 [16, 17]. Therefore, as an additional measure of SIRT1 deacetylase activity, we quantified acetylated protein content of these specific proteins in the same representative sample of 9 cell sets described above at day 9 of adipogenic differentiation. Total acetylated protein was immunoprecipitated from cell supernatant using an antibody to acetylated lysine as described above and the content of PPARγ and β-catenin in the immunoprecipitant was measured on WES, and expressed relative to total PPARɣ and β-catenin protein that was also measured on WES. Antibody specifics for WES are listed in S2 Table. Total acetylated protein was normalized to total protein, as measured by WES, in the respective cell sets.

### Quantitative PCR Analysis

Cells were rinsed with PBS (x2) and then harvested in Buffer RLT (Qiagen, Valencia, CA) with 1% 2-mercaptoethanol (Bio-Rad, Hercules, CA, USA). Total RNA was isolated using RNeasy Plus mini kit (Qiagen, Valencia, CA, USA). Total RNA (0.5 μg) was reverse transcribed using the iScript cDNA synthesis kit (Bio-Rad). Quantitative PCR was performed using primer sets for genes of interest and reference genes (designed using NCBI’s Primer3/BLAST) and iTaq Universal SYBR Green Supermix (Bio-Rad) following manufacturer's protocols. Reactions were run in duplicate on an iQ5 Real Time PCR detection system (Bio-Rad) along with a no-template control per gene. Validation experiments were performed to demonstrate that efficiencies of target and reference genes were approximately equal. Data were normalized to three reference genes using the comparative Ct method. Gene names and primer sequences are listed in S3 Table.

### Statistical Analysis

All analyses were conducted with Type I error rate set to 0.05, using SAS 9.4 (SAS Institute, Cary, North Carolina).

We conducted MANOVA by fitting a separate general linear multivariate model for each protein marker (day 21 PPARγ, SIRT1, and FABP4). The four treatment conditions (vehicle-control, +NAM/-lipid, -NAM/+lipid, +NAM/+lipid) correspond to the elements of a complete two-by-two factorial design. Thus, we tested for the effect of NAM across lipid conditions (main effect of NAM), the effect of lipid across NAM conditions (main effect of lipid), and the interaction between NAM and lipid. Specifically, within the MANOVA we used the Hotelling-Lawley trace to consider the NAM by lipid interaction. If the interaction was non-significant, we tested the main effect of lipid across NAM conditions and the main effect of NAM across lipid conditions. We did not account for multiple comparisons, because we conducted a planned sequence of *a priori* hypothesis tests for only three protein outcomes. We used regression diagnostics to examine the assumptions of multivariate normality of the residuals.

In our sub-sample (N = 9) of the full dataset (N = 46), we used the Wilcoxon signed rank t-test for matched pairs to compare vehicle-control to +NAM/-lipid for the outcomes of ORO, SIRT1 activity, acetylated SIRT1 target proteins, and gene expression.

The percent change in PPARγ protein content in response to +NAM/-lipid treatment was expressed relative to the vehicle-control cells and defined as 100% times the ratio of the mean difference in PPARγ protein content between the +NAM/-lipid condition and the vehicle-control condition (numerator), over the mean PPARγ protein content in the vehicle-control (denominator). The percent increase for FABP4 was calculated similarly.

To examine the association between adipogenic response in the MSCs and the outcome of %FM at birth in the neonate, we fit a general linear univariate model. The two predictors were percent increase in PPARγ and FABP4. We produced 95% confidence intervals for the beta coefficients of the models, as measures of the association between adipogenic response to NAM treatment and adiposity in the neonate.

All data will be made available upon request to the authors.

## Results

### NAM Increased PPARγ on Day 9 in MSCs

**Fig 1:**
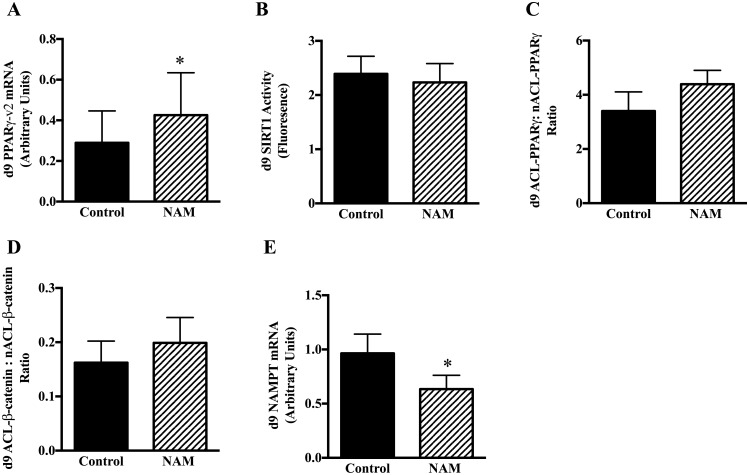
Effects of NAM (3mM) incubation during adipocyte cell differentiation in human MSC. Cells were harvested at day 9 of differentiation and mRNA expression of PPARγ (A), SIRT1 enzyme activity (B), and acetylation of SIRT1 protein targets, PPARγ (C) and β-catenin (D), and mRNA expression of NAMPT (E) in the vehicle-control and NAM only conditions as described in Methods. N = 9 per group. *p<0.05 vs. vehicle-control.

We investigated the effects of NAM at day 9 of differentiation, and as expected, we observed a significant increase in PPARγ mRNA content between the vehicle-control and NAM conditions (+47%, p <0.01) (Fig 1A). SIRT1 activity was unchanged between the vehicle-control treated cells and the NAM-treated cells on day 9 of adipogenic differentiation (Fig 1B) as were levels of acetylated PPARγ and β-catenin (Fig 1C and 1D). However, interestingly, NAMPT mRNA, the rate limiting enzyme in the NAD+ salvage pathway that normally increases SIRT1 activity [18] was significantly lower in the NAM-treated cells compared to the vehicle-control treated cells (-34%, p <0.01) (Fig 1E).

### NAM Incubation Decreased SIRT1 Activity in Differentiated MSCs

**Fig 2:**
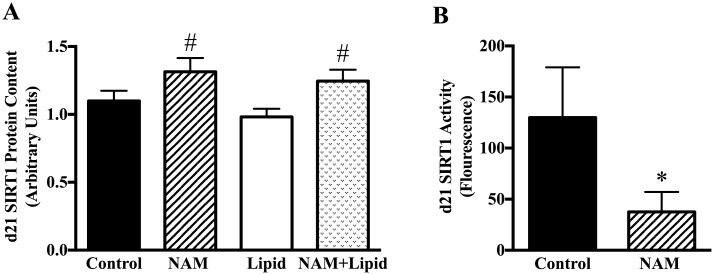
Effects of NAM (3mM) and lipid (200uM) during adipose differentiation on SIRT1 protein and enzyme activity. SIRT1 protein content in MSCs measured by ICE assay at day 21 in all four treatment conditions in the full experimental cohort of 46 cell sets (A). A representative sample of 9 sets of cells from the 46 experimental sets was used for SIRT1 enzyme activity at day 21 in vehicle-control and NAM conditions only (B). *p<0.05 for t-test of NAM vs. vehicle-control; #p<0.05 for main effect of NAM regardless of lipid treatment using MANOVA.

We compared the effect of NAM and lipid treatments over 21 days of differentiation, with the hypothesis that this would decrease SIRT1 protein content. There was no significant interaction between lipid and NAM (p = 0.51). However, there was a significant main effect of NAM on SIRT1 protein (+20%, p<0.01) across lipid conditions (Fig 2A). However, SIRT1 enzyme activity was suppressed significantly at day 21 of differentiation in NAM-treated cells by an average of 71% (p = 0.01) (Fig 2B).

### NAM but Not Lipid Increases Adipogenic Markers in Terminally Differentiated MSCs

**Fig 3:**
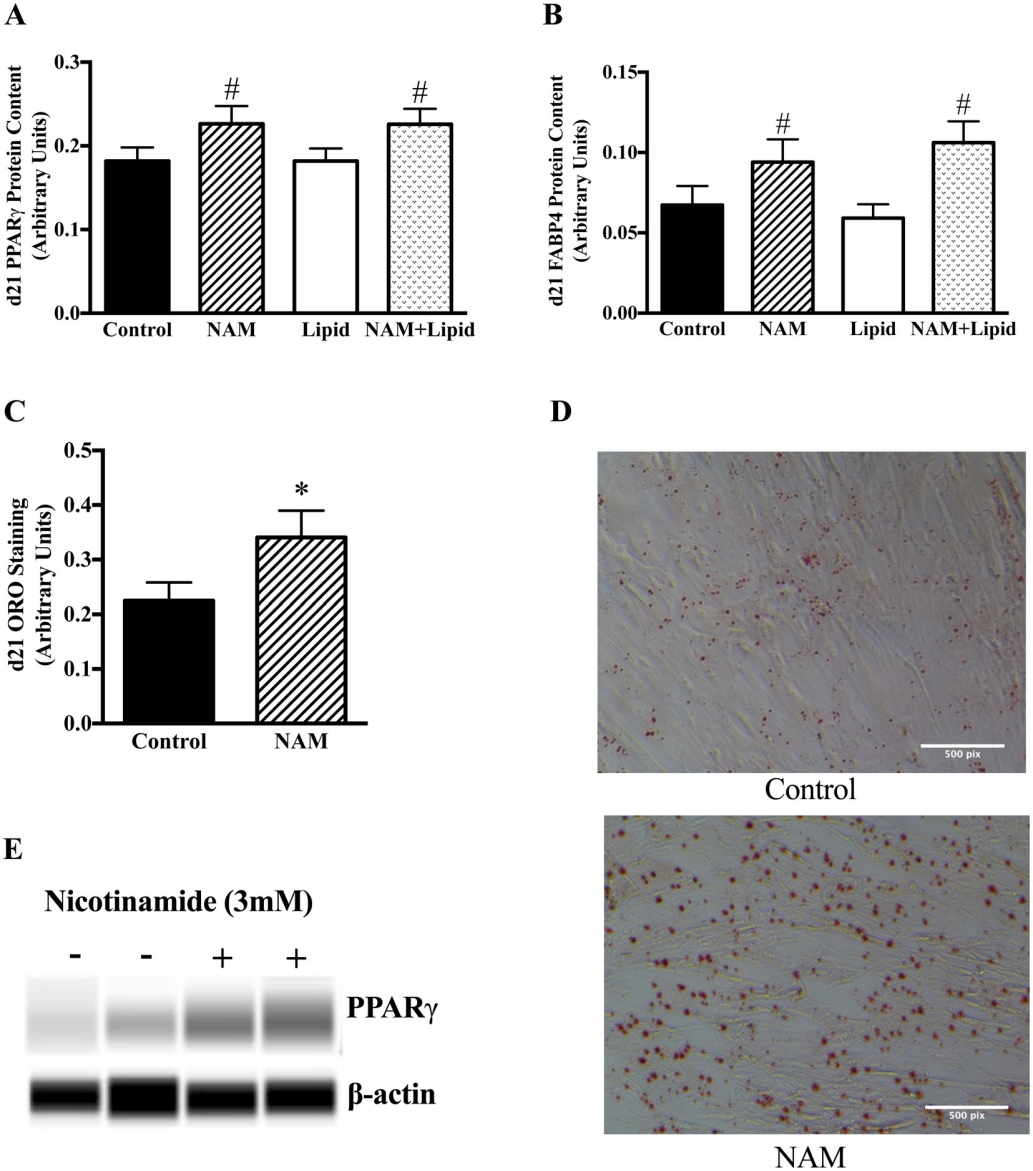
NAM increases PPARγ, FABP4, and lipid content in MSC-derived adipocytes. MSCs were incubated with standard differentiation media for 21 days +/-NAM (3mM) as described in Methods. Protein content at day 21 was measured by ICE assay in all treatment conditions (A and B) (N = 46). *p<0.05 for t-test of NAM vs. vehicle-control; #p<0.05 for main effect of NAM regardless of lipid treatment using MANOVA. A representative sample of 10 sets of cells from the 46 experimental sets was used for day 21 Oil-red O staining (ORO); lipid accumulation measured by ORO in vehicle-control and NAM conditions only and normalized to total protein content (C and D); the scale bar represents 500 pixels. Photographs were taken with a 10x objective lens on a phase contrast microscope. Photographs are for representative purposes only; quantitative assessment of ORO staining was obtained using spectrophotometric measures. A representative sample of PPARγ protein as measured by WES is shown in (E).

To test the effects of lipid and NAM on differentiation, we measured PPARγ and its down-stream target FABP4 on day 21 of differentiation. The interaction between NAM and lipid treatment was non-significant for PPARγ and FABP4 protein (p = 0.99 and p = 0.09 for each, respectively). When testing for the main effects we found that NAM treated cells had significantly greater PPARγ and FABP4 protein content regardless of lipid treatment (PPARγ +24%, p <0.01; FABP4 +57%, p <0.01), whereas there was no significant main effect of lipid on either (p = 0.98 and p = 0.82 for each, respectively) (Fig 3A and 3B). Further, lipid accumulation, as measured by ORO was significantly greater in cells treated with NAM, compared to vehicle-control (+51%, p <0.05) as shown in Fig 3C and 3D.

### Adipogenic Response to NAM in MSCs is Associated with Infant Adiposity at Birth

**Fig 4:**
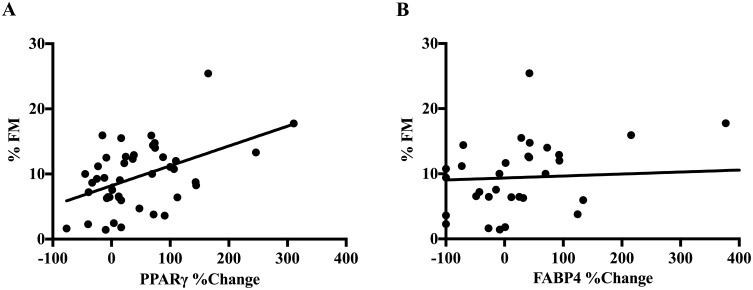
Scatter plots depicting the correlation between neonatal adiposity and the percent change of PPARγ (A) and FABP4 (B) protein content at day 21 between vehicle-control and NAM only conditions (N = 46).

We compared the NAM-induced adipogenic response in MSCs with the %FM of the neonate from which the cells were derived (Fig 4). In investigating the independent associations between PPARγ and FABP4, we found that the percent change in PPARγ protein between the NAM (+NAM/-lipid) and vehicle-control condition was significantly and independently associated with neonatal %FM at birth (β = 0.04, 95% CI 0.01–0.06, p <0.001) while the percent increase in FABP4 protein was not associated with %FM (p = 0.53) when adjusted by PPARγ.

## Discussion

The present study tested the hypothesis that SIRT1 may be involved in micronutrient-induced enhancement of adipogenic differentiation of human MSCs. Our results suggest that NAM inhibits SIRT1’s activity during adipogenic differentiation and increases adipogenic protein markers in MSCs. While this is the first study to demonstrate this effect in human MSCs, our results are consistent with other cell culture studies of the effect of SIRT1 inhibitors on adipogenic cell fate using different species and cell lines [5, 19].

We have also shown in this study that lipid exposure did not increase adipogenic protein markers in our MSCs; rather NAM exposure appeared to account entirely for the increased adipogenic response in the NAM-only and co-incubated NAM and lipid conditions. Intracellular lipid accumulation in the NAM condition was also significantly higher than the vehicle-control condition. Together these results suggest that NAM may accelerate adipogenesis even in the absence of lipids. However, whether NAM is capable of inducing adipogenesis alone, without concomitant adipogenic induction, is not known. Further, our failure to induce greater adipogenesis with lipid treatment may be due in part to our attempt at mimicking a physiologically relevant fatty acid concentration ratio of oleate-to-palmitate [15, 20] whereas other studies of fatty acid-induced adipogenesis have used much higher lipid doses [21, 22].

Our study showed a potentially delayed effect of chronic exogenous NAM exposure on reducing SIRT1 action during adipogenesis in that SIRT1 activity was not significantly reduced until terminal adipogenic differentiation (day 21). Our early analysis on day 9 showed that NAM treatment appeared to reduce the key NAD+ salvage pathway enzyme, NAMPT. The intracellular *de-novo* synthesis of NAD+ from NAM is dependent on the NAD+ Salvage Pathway that involves the rate-limiting enzyme, NAMPT. The availability and activity of NAMPT is essential for maintaining the NAD+-to-NAM ratio and is therefore important for regulating SIRT1 activity [18, 23, 24]. In a murine model of MSC cell fate, Yi and colleagues demonstrated that the NAMPT inhibitor, FK866, increased intracellular NAM levels and decreased SIRT1 activity which subsequently increased PPARγ expression, lipid accumulation, and adipocyte formation [19].

In our experimental model, the early reduction in NAMPT expression would potentially diminish the intracellular pool of NAD+ and increase the endogenous levels of NAM. This decreased NAD-to-NAM ratio could compound the direct inhibitory effects of the exogenous NAM, possibly reaching a threshold later in the adipogenic induction, which could explain why SIRT1 activity was not significantly lower in the NAM condition until day 21 of adipogenic differentiation. There was a slightly higher SIRT1 protein content at day 21 in the NAM conditions despite significantly reduced SIRT1 enzyme activity (+70%) at this same time point. This discordance may be due, in part, to a compensatory response of the SIRT1 enzyme to the reduced availability of the NAD+ substrate for enzyme activity possibly resulting from the early (day 9) reduction in NAMPT. However, in several studies NAMPT was reportedly increased during adipogenic induction of 3T3-L1 cells [22, 25], therefore suggesting that under normal adipogenic conditions NAMPT is up-regulated presumably to accommodate the increased demand for NAD+. It remains unknown, however, how NAM directly impacts NAMPT expression and activity and therefore this finding and proposed mechanism requires further investigation. Further, the increase in PPARγ in the early phases of differentiation without a change in SIRT1 activity suggests that the effects of NAM on PPARγ expression in early adipogenesis are not straightforward, and could be governed by other early factors that affect PPARγ expression (e.g. C/EBPs, CREB, or OCT1).

Importantly, this is the first study to demonstrate a significant association between *in-vitro* markers of adipogenesis in the MSCs treated with NAM and *in-vivo* neonatal adiposity at birth in the infants from which the cells were derived; infants with greater adiposity at birth had correspondingly larger percent changes for cellular PPARγ in response to NAM treatment. This finding, notably in cells derived from infants born to non-obese mothers, suggests a potential adipogenic susceptibility of MSCs to excess NAM exposure in-utero. Further, given that the MSC population is retained throughout an individual’s lifetime, this MSC model of adipogenesis and NAM exposure also highlights the potential for postnatal sensitivity to dietary NAM-enhanced adipogenesis.

In addition to its novelty, our study has several significant strengths. Most importantly is the relevance of the MSC model for studying in-utero and postnatal adipogenic mechanisms in human offspring given that they are sourced directly from fetal tissue and are precursor cells to adipose tissue as well as other metabolically active tissues. Furthermore, the parent study, Healthy Start provides extensive phenotyping of participants both during pregnancy and postnatally. This has facilitated our ability to take *in-vitro* measures of adipogenesis and compare them to *in-vivo* adiposity of the participating infants, therefore helping to generalize our findings to living, intact human systems. The detailed information available from Healthy Start will also allow future mechanistic investigations using the BabyBUMP Project sample into the impact of other pregnancy and postnatal exposures on fat, muscle, bone, and some neuronal tissue development.

In conclusion, our study has shown the potentially “obesogenic” effect of excess NAM *in-vitro*. NAM is ubiquitous in the diet and may be even more prevalent in diets with high consumption of processed grains and animal products. Among pregnant women specifically, additional NAM, and not nicotinic acid is consumed through prenatal vitamins [26]. In the context of our *in-vitro* results presented here, further investigation is needed to determine whether maternal NAM consumption during pregnancy in a free-living human population contributes to greater infant adiposity at birth.

## Supporting Information

S1 Fig A dose-response curve of NAM (2.5, 3.0, 4.5 mM) was tested to determine the optimal concentration of NAM to increase PPARγ protein content on day 21 of differentiation as a marker of increased adipogenic response. For all experiments we used 3 mM NAM.
(EPS)

S1 Table Adipogenic differentiation media recipes and induction schedule. (DOCX)

S2 Table Antibodies and their dilutions for the differentprotein detection methods used in this study. (DOCX)

S3 Table Gene names and primer sequences for PCR analysis. (DOCX)

## References

[1] Ahmed T, Hossain M, Sanin KI. Global burden of maternal and child undernutrition and micronutrient deficiencies. Annals of nutrition & metabolism. 2012;61 Suppl 1:8–17. 23343943 10.1159/000345165

[2] Krasnow SM, Nguyen MLT, Marks DL. Increased maternal fat consumption during pregnancy alters body composition in neonatal mice. American journal of physiology Endocrinology and metabolism. 2011;301:E1243–53. 10.1152/ajpendo.00261.2011 21900122 PMC3233776

[3] Rebholz SL, Burke KT, Yang Q, Tso P, Woollett LA. Dietary fat impacts fetal growth and metabolism: uptake of chylomicron remnant core lipids by the placenta. American journal of physiology Endocrinology and metabolism. 2011;301:E416–25. 10.1152/ajpendo.00619.2010 21586694 PMC3154537

[4] Grant WF, Gillingham MB, Batra AK, Fewkes NM, Comstock SM, Takahashi D, et al Maternal high fat diet is associated with decreased plasma n-3 fatty acids and fetal hepatic apoptosis in nonhuman primates. PloS one. 2011;6(2):e17261 10.1371/journal.pone.0017261 21364873 PMC3045408

[5] Bäckesjö C-M, Li Y, Lindgren U, Haldosén L-A. Activation of Sirt1 decreases adipocyte formation during osteoblast differentiation of mesenchymal stem cells. Journal of bone and mineral research: the official journal of the American Society for Bone and Mineral Research. 2006;21:993–1002. 10.1359/jbmr.060415 16813520

[6] Goldie C, Taylor AJ, Nguyen P, McCoy C, Zhao XQ, Preiss D. Niacin therapy and the risk of new-onset diabetes: a meta-analysis of randomised controlled trials. Heart (British Cardiac Society). 2016;102(3):198–203. 26370223 10.1136/heartjnl-2015-308055 PMC4752613

[7] Picard F, Kurtev M, Chung N, Topark-Ngarm A, Senawong T, Machado De Oliveira R, et al Sirt1 promotes fat mobilization in white adipocytes by repressing PPAR-gamma. Nature. 2004;429:771–6. 15175761 10.1038/nature02583 PMC2820247

[8] Mayoral R, Osborn O, McNelis J, Johnson AM, Oh da Y, Izquierdo CL, et al Adipocyte SIRT1 knockout promotes PPARgamma activity, adipogenesis and insulin sensitivity in chronic-HFD and obesity. Molecular metabolism. 2015;4(5):378–91. 10.1016/j.molmet.2015.02.007 25973386 PMC4421024

[9] Fischer-Posovszky P, Kukulus V, Tews D, Unterkircher T, Debatin K-M, Fulda S, et al Resveratrol regulates human adipocyte number and function in a Sirt1-dependent manner. The American journal of clinical nutrition. 2010;92:5–15. 10.3945/ajcn.2009.28435 20463039

[10] Chalkiadaki A, Guarente L. High-fat diet triggers inflammation-induced cleavage of SIRT1 in adipose tissue to promote metabolic dysfunction. Cell metabolism. 2012;16:180–8. 10.1016/j.cmet.2012.07.003 22883230 PMC3539750

[11] Suter MA, Chen A, Burdine MS, Choudhury M, Harris RA, Lane RH, et al A maternal high-fat diet modulates fetal SIRT1 histone and protein deacetylase activity in nonhuman primates. FASEB journal: official publication of the Federation of American Societies for Experimental Biology. 2012;26:5106–14. 22982377 10.1096/fj.12-212878 PMC3509051

[12] Starling AP, Brinton JT, Glueck DH, Shapiro AL, Harrod CS, Lynch AM, et al Associations of maternal BMI and gestational weight gain with neonatal adiposity in the Healthy Start study. The American journal of clinical nutrition. 2015;101:302–9. 10.3945/ajcn.114.094946 25646327 PMC4307203

[13] Boyle KE, Patinkin ZW, Shapiro AL, Baker PR 2nd, Dabelea D, Friedman JE. Mesenchymal Stem Cells From Infants Born to Obese Mothers Exhibit Greater Potential for Adipogenesis: The Healthy Start BabyBUMP Project. Diabetes. 2016;65(3):647–59. 10.2337/db15-0849 26631736 PMC4764150

[14] Janderova L, McNeil M, Murrell AN, Mynatt RL, Smith SR. Human mesenchymal stem cells as an in vitro model for human adipogenesis. Obesity research. 2003;11(1):65–74. 12529487 10.1038/oby.2003.11

[15] Koves TR, Ussher JR, Noland RC, Slentz D, Mosedale M, Ilkayeva O, et al Mitochondrial overload and incomplete fatty acid oxidation contribute to skeletal muscle insulin resistance. Cell Metab. 2008;7(1):45–56. 10.1016/j.cmet.2007.10.013 18177724

[16] Han L, Zhou R, Niu J, McNutt MA, Wang P, Tong T. SIRT1 is regulated by a PPAR{γ}-SIRT1 negative feedback loop associated with senescence. Nucleic acids research. 2010;38:7458–71. 10.1093/nar/gkq609 20660480 PMC2995042

[17] Simic P, Zainabadi K, Bell E, Sykes DB, Saez B, Lotinun S, et al SIRT1 regulates differentiation of mesenchymal stem cells by deacetylating β-catenin. EMBO molecular medicine. 2013;5:430–40. 10.1002/emmm.201201606 23364955 PMC3598082

[18] Zhang T, Berrocal JG, Frizzell KM, Gamble MJ, DuMond ME, Krishnakumar R, et al Enzymes in the NAD+ salvage pathway regulate SIRT1 activity at target gene promoters. The Journal of biological chemistry. 2009;284(30):20408–17. 10.1074/jbc.M109.016469 19478080 PMC2740465

[19] Zeljkovic A, Vekic J, Spasic S, Jelic-Ivanovic Z, Spasojevic-Kalimanovska V, Gojkovic T, et al Changes in LDL and HDL subclasses in normal pregnancy and associations with birth weight, birth length and head circumference. Maternal and child health journal. 2013;17:556–65. 10.1007/s10995-012-1031-x 22527773

[20] Maples JM, Brault JJ, Witczak CA, Park S, Hubal MJ, Weber TM, et al Differential epigenetic and transcriptional response of the skeletal muscle carnitine palmitoyltransferase 1B (CPT1B) gene to lipid exposure with obesity. Am J Physiol Endocrinol Metab. 2015;309(4):E345–56. 10.1152/ajpendo.00505.2014 26058865 PMC4537922

[21] Petrighi Polidori G, Lomax MA, Docherty K. Palmitate enhances the differentiation of mouse embryonic stem cells towards white adipocyte lineages. Mol Cell Endocrinol. 2012;361(1–2):40–50. 10.1016/j.mce.2012.03.010 22484460

[22] Sasaki T, Maier B, Koclega KD, Chruszcz M, Gluba W, Stukenberg PT, et al Phosphorylation regulates SIRT1 function. PloS one. 2008;3:e4020 10.1371/journal.pone.0004020 19107194 PMC2602979

[23] Burgos ES. NAMPT in regulated NAD biosynthesis and its pivotal role in human metabolism. Current medicinal chemistry. 2011;18:1947–61. 21517777 10.2174/092986711795590101

[24] Song TY, Yeh SL, Hu ML, Chen MY, Yang NC. A Nampt inhibitor FK866 mimics vitamin B3 deficiency by causing senescence of human fibroblastic Hs68 cells via attenuation of NAD(+)-SIRT1 signaling. Biogerontology. 2015;16(6):789–800. 10.1007/s10522-015-9605-9 26330291

[25] Kralisch S, Klein J, Lossner U, Bluher M, Paschke R, Stumvoll M, et al Hormonal regulation of the novel adipocytokine visfatin in 3T3-L1 adipocytes. The Journal of endocrinology. 2005;185(3):R1–8. 15930160 10.1677/joe.1.06211

[26] U.S. National Library of Medicine. Dietary Supplements Labels Database. Available: http://dietarysupplements.nlm.nih.gov/dietary on August 8, 2015.

