# Supplementary material for: Nicotinamide Promotes Adipogenesis in Umbilical Cord-Derived Mesenchymal Stem Cells and Is Associated with Neonatal Adiposity: The Healthy Start BabyBUMP Project
Source: PLoS One. 2016 Jul 14;11(7):e0159575. doi: 10.1371/journal.pone.0159575 (PMC4944979; doi:10.1371/journal.pone.0159575)
Supplement: S1 Table — (DOCX) [file pone.0159575.s002.docx]

**S1 Table**. Adipogenic differentiation media

recipes and induction schedule.

| Experiment Day | Media | Media Ingredients |
| --- | --- | --- |
| 0-2, 6-8, 12-14 | AIM | low-glucose DMEM  5% FBS  0.01% PEN-STREP  1.0 uM DEX  0.2 mM INDO  170 nM insulin |
| 3-5, 9-11, 15-21 | AMM | low-glucose DMEM  5% FBS  0.01% PEN-STREP  170 nM insulin |
