# Supplementary material for: Nicotinamide Promotes Adipogenesis in Umbilical Cord-Derived Mesenchymal Stem Cells and Is Associated with Neonatal Adiposity: The Healthy Start BabyBUMP Project
Source: PLoS One. 2016 Jul 14;11(7):e0159575. doi: 10.1371/journal.pone.0159575 (PMC4944979; doi:10.1371/journal.pone.0159575)
Supplement: S2 Table — (DOCX) [file pone.0159575.s003.docx]

**S2 Table.** Antibodies and their dilutions for the different

protein detection methods used in this study.

| Detection Method | Primary  Antibody | Dilution Used | Secondary Antibody | Dilution Used |
| --- | --- | --- | --- | --- |
| ICE | PPARγ | 1:200 | Goat≠Rabbit | 1:7,500 |
|  | FABP4 | 1:1,000 |  | 1:5,000 |
|  | SIRT1 | 1:250 |  | 1:2,500 |
|  | β-actin | 1:200 |  | 1:5,000 |
| WES | PPARγ | 1:25 | Goat≠Rabbit | 1:50 |
|  | β-catenin | 1:50 |  | 1:50 |
