# Supplementary material for: Nicotinamide Promotes Adipogenesis in Umbilical Cord-Derived Mesenchymal Stem Cells and Is Associated with Neonatal Adiposity: The Healthy Start BabyBUMP Project
Source: PLoS One. 2016 Jul 14;11(7):e0159575. doi: 10.1371/journal.pone.0159575 (PMC4944979; doi:10.1371/journal.pone.0159575)
Supplement: S3 Table — (DOCX) [file pone.0159575.s004.docx]

**S3 Table.** Gene names and primer sequences for PCR analysis.

| Gene Name | 5’ Primer | 3’ Primer |
| --- | --- | --- |
| PPARγ v2 | AGCAAACCCCTATTCCATGCT | TGTGTCAACCATGGTCATTTCTTG |
| NAMPT | GTGGAGGTTTGCTACAGAAGT | TGGGTCCTTGAAGACGTTAATC |
| RLP13A (ref.) | CCTGGAGGAGAAGAGGAAAGAGA | TTGAGGACCTCTGTGTATTTGTCAA |
| ACTB (ref.) | CACTCTTCCAGCCTTCCTTC | GTACAGGTCTTTGCGGATGT |
